# Optimizing TILLING and Ecotilling techniques for potato (*Solanum tuberosum *L)

**DOI:** 10.1186/1756-0500-2-141

**Published:** 2009-07-17

**Authors:** Rana Elias, Bradley J Till, Chikelu Mba, Bassam Al-Safadi

**Affiliations:** 1 Atomic Energy Commission of Syria, PO. Box 6091, Damascus, Syria; 2 Plant Breeding Unit, FAO/IAEA Agricultural & Biotechnology Laboratory, International Atomic Energy Agency, P.O. Box 100, Wagramer Strasse 5, A-1400 Vienna, Austria

## Abstract

**Background:**

The TILLING and Ecotilling techniques for the discovery of nucleotide polymorphisms were applied to three potato (*Solanum tuberosum*) cultivars treated with gamma irradiation. The three mutant cultivars tested were previously shown to exhibit salinity tolerance, an important trait in countries like Syria where increasing soil salinity is affecting agricultural production.

**Findings:**

Three gene-specific primer pairs were designed from BAC sequence to amplify ~1 to 1.5 kb of gene target. One of the three primer pairs amplified a single gene target. We used this primer pair to optimize enzymatic mismatch cleavage and fluorescence DNA detection for polymorphism discovery. We identified 15 putative nucleotide polymorphisms per kilobase. Nine discovered polymorphisms were unique to one of the three tetraploid cultivars tested.

**Conclusion:**

This work shows the utility of enzymatic mismatch cleavage for TILLING and Ecotilling in different varieties of potato. The method allows for rapid germplasm characterization without the cost and high informatics load of DNA sequencing. It is also suitable for mutation discovery in high-throughput reverse genetic screens.

## Background

Potato is considered the fourth most important food crop in the world due to its high productivity and nutritional value. Globally, potato production is hindered by a number of biotic and abiotic stresses. In Syria, a major stress on agricultural output of potato is increasing soil salinity. Sterility and tetraploidy in conjunction with a high level of heterozygosity greatly reduce the ability to use traditional methods of breeding, specifically hybridization and selection, for potato improvement. Mutagenesis in potato has been used as an alternative approach for generating nucleotide diversity and inducing useful traits. This approach, usually referred to as mutation breeding, has been used in developing several salinity tolerant and disease resistant potato lines in Syria [1,2].

Reverse genetic strategies focus on the identification of lesions in specific genes prior to any phenotypic characterization. This takes advantage of the growing knowledge of genomic sequence and hypotheses for gene functions based on research in model organisms and multiple sequence alignments. TILLING (Targeting Induced Local Lesions IN Genomes) is a general reverse genetic technique that combines traditional mutagenesis with high-throughput mutation discovery [3,4]. It allows the discovery of an allelic series of induced point mutations in candidate genes in a collection of mutagenized individuals. TILLING has been applied successfully in plants and animals, and collectively, the discovery of thousands of mutations has been described [5].

The most commonly used strategy for mutation discovery in TILLING is enzymatic mismatch cleavage followed by fluorescence detection using the LI-COR DNA analyzer [6]. Target DNA of ~1.5 kb is end-labeled by PCR using fluorescently labeled primers containing the IRDye 700 (forward) and IRDye 800 (reverse) dyes. After PCR, samples are denatured and annealed to form heteroduplexes between wild type and polymorphic amplicons. Mismatches are cleaved by incubation with a single-strand specific nuclease. Products are size-fractionated by denaturing polyacrylamide gel electrophoresis and visualized by fluorescence detection using the LI-COR instrument. In addition to being used for the detection of induced mutation events, the method can also be used to discover natural nucleotide differences in plant or animal populations, a process called Ecotilling because it was first used to study natural diversity in different Arabidopsis ecotypes [7].

The goal of this work was to establish optimized protocols for primer deign, testing and CEL I/LI-COR based TILLING and Ecotilling screens using potato genomic DNAs. We have identified optimal conditions for the discovery of unique nucleotide polymorphisms in three potato cultivars. This work shows the utility of TILLING and Ecotilling strategies in potato and provides a foundation for larger scale efforts in reverse genetics and characterization of natural nucleotide variation.

## Methods and results

Three locally grown varieties of potato (Draja, Sponta, and Diamond) from a previously conducted mutation breeding program for salinity tolerance were used in the study [2]. Genomic DNAs were prepared using the DNeasy plant mini kit (QIAGEN). DNAs were evaluated for quality and quantity by agarose gel electrophoresis (Additional file 1). Two replicates (siblings showing the same phenotype) of the Draja, Sponta, and Diamond varieties were chosen for assay optimization, for a total of six unique samples. Each sample was normalized to a concentration of 20 ng/μl in TE buffer. Gene-specific primers were designed using BAC sequence from the Potato Genome Sequencing Consortium [8]. Primers were designed using parameters previously described for TILLING (Additional file 2, [6]).

We used a two-phase testing approach for primer validation: amplicon analysis by agarose gel electrophoresis, followed by sequence analysis of amplicons. Primers that pass the agarose gel phase produce a single amplicon of the expected molecular weight that is at a concentration of >= 5 ng/μl. Primer pairs passing the second phase produce good quality sequence trace data showing the amplification of only one gene product with ~<= 5% "heterozygous" polymorphisms as observed by overlapping ABI sequence trace peaks (Additional file 3). Primer pair st_3_till passed both testing phases for a total of 33% (1/3) passing primer sequences. The 905 bp amplicon is homologous to a *Solanum tuberosum *cDNA clone (Genbank accession EG015453), but no significant homologies to other species were discovered in nucleotide BLAST searches of Genbank. While this amplicon is likely not suitable for the discovery of the causative mutation(s) of salinity tolerance, it was deemed suitable for validating TILLING and Ecotilling in potato.

**Figure 1:**
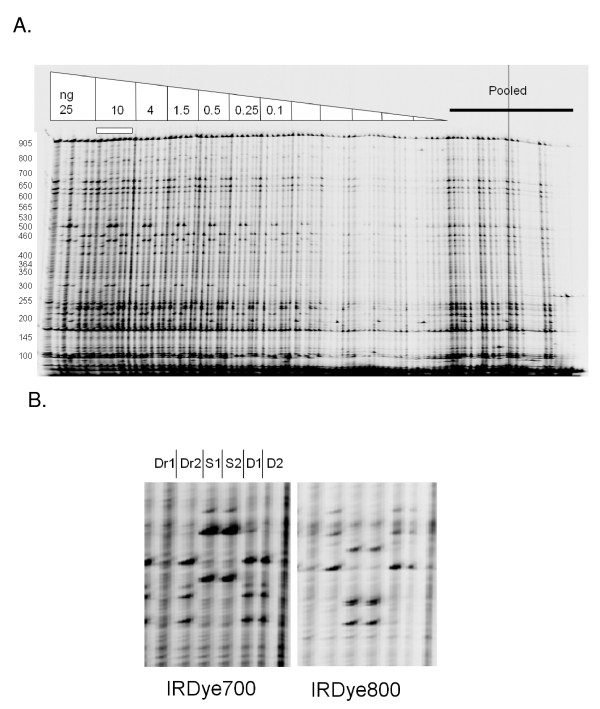
**Enzymatic mismatch cleavage and fluorescence detection of potato polymorphisms**. IRDye 700 image shown for optimization of input genomic DNA for PCR amplification (A). DNA amounts of 25, 10, 4, 1.5, 0.5, 0.25, 0.1, 0.04, 0.03, 0.015, 0.025 and 0.01 ng were tested in PCR reactions with fluorescently labeled primers (listed on top of image). After PCR, samples were denatured and annealed to form heteroduplexes between polymorphic amplicons. Mismatches were cleaved by incubating samples with a crude celery extract containing the CEL I enzyme. Samples were resolved by denaturing polyacrylamide gel electrophoresis and visualized with the LI-COR DNA analyzer. Bands represent nucleotide polymorphisms. Molecular weights estimated using the GelBuddy program [9] are listed to the left of the image. Polymorphisms were also detected in pooled samples. At each DNA concentration, two samples of the Draja (Dr), Sponta (S) and Diamond (D) genotypes were tested (Panel B, image from lanes marked with a horizontal box in panel A). True polymorphisms produce a band in the IRDye700 image channel and a band in the IRDye800 image channel whose molecular weights sum to the weight of the full length PCR product. The high background and low band resolution observed in sample Draja1 is indicative of genomic DNA degradation.

Genomic DNAs from each genotype were arrayed in a 96 well format at varying DNA concentrations starting at 5 ng/μl to 0.0002 ng/μl. Samples from each genotype were also mixed together to test the effect of pooling samples on polymorphism discovery. PCR amplification with genomic DNA at varying concentrations, primer pair st_3, mismatch cleavage using celery juice extract, gel eletrophoresis and image analysis followed previously described protocols [6]. The resulting gel image showed strong signal in PCR reactions using genomic DNA amounts between 25 and 0.25 ng (Figure 1, [9]).

Previous studies have shown that band analysis on LI-COR gels is a highly accurate, low cost and robust method for discovering novel nucleotide diversity including SNPs and small indels [7,10]. We chose to analyze bands between positions 800 and 200 bp from the forward primer binding site of the amplicon because this region provided the best signal to noise for manual image analysis using the GelBuddy program (Table 1). Each band discovered in the IRDye700 channel had a complementary band in the IRDye800 channel whose molecular weights when added was similar to the molecular weight of the full length PCR product. The same putative polymorphisms were discovered using both samples of the Sponta genotype. The two Diamond samples and the second Draja sample shared the same bands. In comparison between the two Draja samples, 25% (3/12) bands discovered in Draja2 were not observed in Draja1. Lane quality for Draja1 samples was reduced (Figure 1), and we hypothesize that bands were not detected in Draja1 due to partial degradation of the genomic DNA, possibly due to nicking that was not observable during native agarose gel electrophoresis (Additional file 1). We identified no bands unique to either Draja or Diamond samples as would be expected from an induced mutation from gamma irradiation treatment. This is not surprising given the scale of this study and what is known about mutation densities in chemically and physically mutagenized diploid and polyploid species. The three mutant lines tested here were first selected based on phenotype and so the population size for genotypic screening was three rather than thousands of individuals typically screened in TILLING applications. With no discovered mutations in Draja or Diamond in an amplicon of 905 bp, we can only estimate that the mutation density is less than 1 mutation per 1810 bp (2 × 905). This upper limit in mutation density is much higher than actual densities reported for tetraploid wheat treated with EMS (1 mutation per 40 kb), or diploid rice treated with gamma irradiation (1/6190 kb, [11,12]). It remains possible that one or more bands identified in the Sponta samples were induced through mutagenesis. This can be determined by screening non-mutagenized samples of the same genotype.

**Table 1:** Band analysis of potato cultivars

MW (bp) IRD700	Draja1	Draja2	Spontna1	Spontna2	Diamond1	Diamond2
797			+	+		

780	+	+			+	+

772			+	+		

686			+	+		

673	+	+			+	+

641	+	+	+	+	+	+

625	+	+			+	+

625			+	+		

563	+	+			+	+

521			+	+		

500			+	+		

466	+	+			+	+

449			+	+		

432		+			+	+

406	+	+			+	+

308			+	+		

286		+			+	+

252	+	+	+	+	+	+

243		+			+	+

236	+	+	+	+	+	+

218			+	+		

Total	9	12	12	12	12	12

From this work we have identified 21 novel potato markers. Nine are unique to the Sponta genotype and nine are common in Draja and Diamond but not found in Sponta samples. No unique markers between Draja and Diamond were discovered. When screening samples individually, only heterozygous polymorphisms are identified. In the 600 base pair window of band analysis, we identified 12 paired bands in all but the Draja1 sample. We therefore estimate that this region is ~2% heterozygous (12/600) for all tested genotypes. No new bands were observed when screening pooled samples suggesting that only heterozygous differences exist between the three cultivars in this genomic region.

## Conclusion

In this pilot study to optimize the TILLING and Ecotilling methods for potato, a range of genomic DNA concentrations between 25 ng and 0.25 ng was identified that can be used for PCR to produce high quality gel images. In testing three primer pairs designed using available BAC sequence a 66% percent failure rate was observed. The high failure rate may be influenced by the low number of tested primers, incomplete genomic sequence and polyploidy in potato. While potentially limiting, primer design failures can be overcome with careful pre-testing as described here. In the present study 21 novel markers that can be used to differentiate between Sponta and the Draja and Diamond genotypes were identified. We therefore conclude that Ecotilling is a suitable technique for the discovery of natural nucleotide variation in potato and that TILLING is a viable method providing a suitably mutagenized population can be prepared.

## Competing interests

The authors declare that they have no competing interests.

## Authors' contributions

RE performed all experiments described and participated in drafting the manuscript. BJT provided experimental design and data analysis, and participated in drafting the manuscript. CM provided project oversight at the Agricultural and Biotechnology Laboratory, contributed to project planning and contributed to the critical revision of the manuscript. BS provided potato varieties used in this study, project oversight at the Atomic Energy Commission of Syria and participated in drafting the manuscript. All authors read and approved the final manuscript.

## Supplementary Material

Additional file 1 **Agarose gel image to estimate intactness and concentration of genomic DNA samples**. The image shows quality and quantity evaluations for fourteen genomic DNA preparations of the Draja variety. Click here for file

Additional file 2 **Primers designed for TILLING assays**. The table provides nucleotide sequences for primers used in this study. Click here for file

Additional file 3 **Agarose gel and sequence evaluation of products from test primers**. The figure shows examples of passing and failing primer pairs as determined by agarose gel and sequence analysis of PCR amplicons. Click here for file
