# Supplementary figures and images for: Optimizing TILLING and Ecotilling techniques for potato (Solanum tuberosum L)
Source: BMC Res Notes. 2009 Jul 17;2:141. doi: 10.1186/1756-0500-2-141 (PMC2717973; doi:10.1186/1756-0500-2-141)

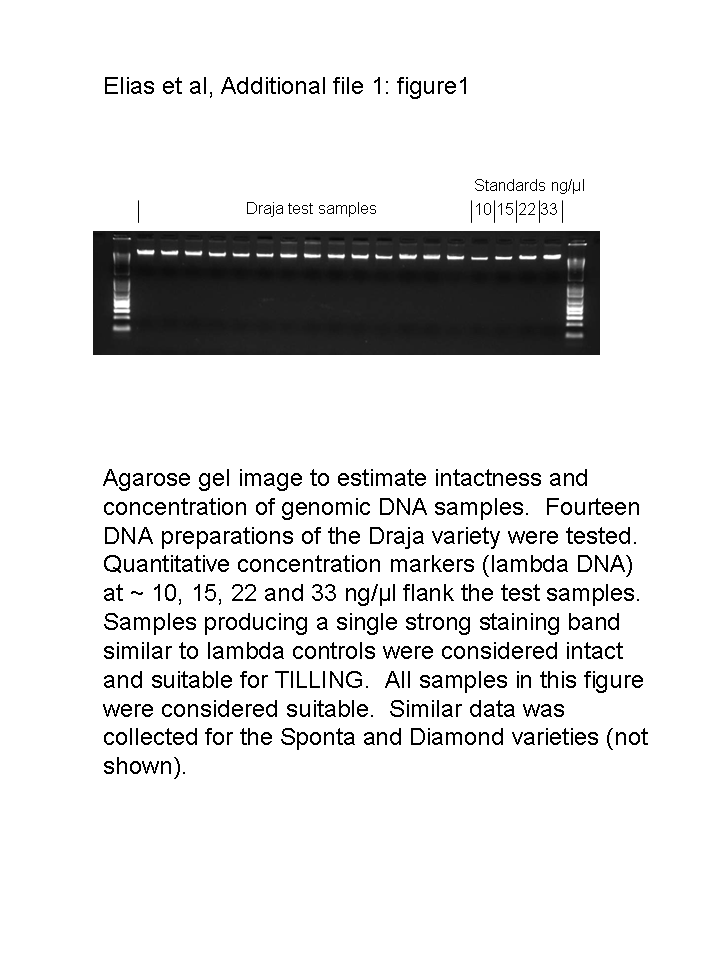

Supplement: Additional file 1 — Agarose gel image to estimate intactness and concentration of genomic DNA samples. The image shows quality and quantity evaluations for fourteen genomic DNA preparations of the Draja variety. [file 1756-0500-2-141-S1.tiff]

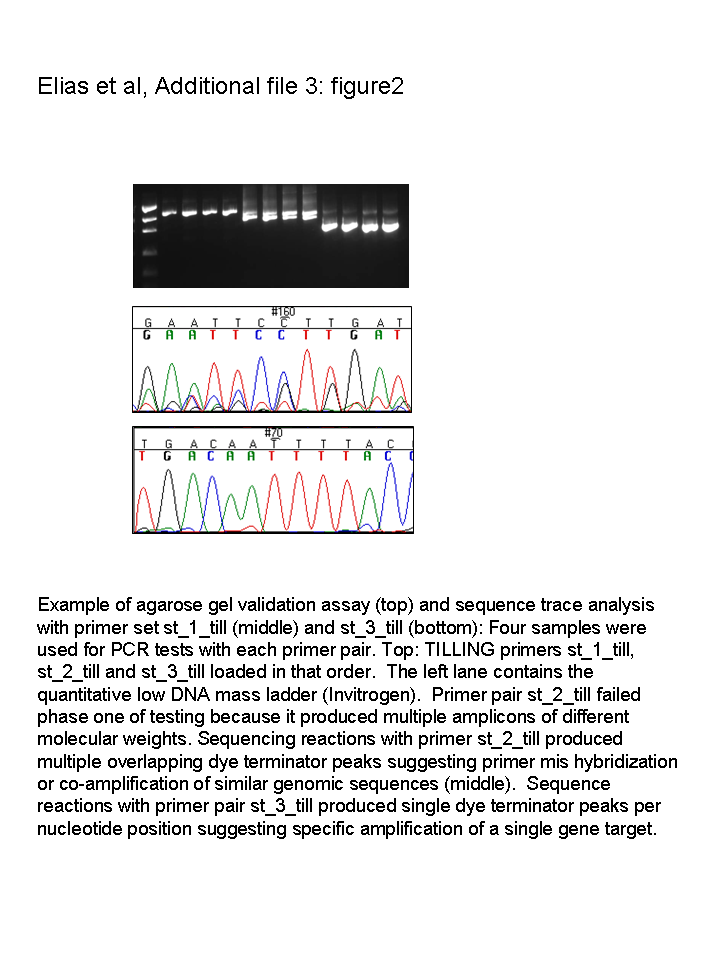

Supplement: Additional file 3 — Agarose gel and sequence evaluation of products from test primers. The figure shows examples of passing and failing primer pairs as determined by agarose gel and sequence analysis of PCR amplicons. [file 1756-0500-2-141-S3.tiff]
