# Supplementary material for: Optimizing TILLING and Ecotilling techniques for potato (Solanum tuberosum L)
Source: BMC Res Notes. 2009 Jul 17;2:141. doi: 10.1186/1756-0500-2-141 (PMC2717973; doi:10.1186/1756-0500-2-141)
Supplement: Additional file 2 — Primers designed for TILLING assays. The table provides nucleotide sequences for primers used in this study. [file 1756-0500-2-141-S2.pdf]

Elias et al, Additional file 2: Additional table 1

Additional Table 1: Primers designed for TILLING assays

**Name**	**Sequence**
st_1_tillL	TCAACAAAGAAAGCTTAGATGCGAAGGA
st_1_tillR	TGAATGAAATCAAAGTAGATGGCTTGCTGA
st_2_tillL	TGCGAAAAAGCGCACAATTGAACA
st_2_tillR	GGGGCCTGGTGAGGGTAGGAATTT
st_3_tillL	GAATTAGGCTCGGGGACCTGTCGT
st_3_tillR	GACTAAAGGCAGAGGCCGGCTGAG
